# Identifying topologically associating domains and subdomains by Gaussian Mixture model And Proportion test

**DOI:** 10.1038/s41467-017-00478-8

**Published:** 2017-09-14

**Authors:** Wenbao Yu, Bing He, Kai Tan

**Affiliations:** 1 Department of Biomedical and Health Informatics, Children’s Hospital of Philadelphia, Philadelphia, PA 19104 USA; 2 Division of Oncology and Center for Childhood Cancer Research, Children’s Hospital of Philadelphia, Philadelphia, PA 19104 USA; 3 Department of Pediatrics, Perelman School of Medicine, University of Pennsylvania, Philadelphia, PA 19104 USA

## Abstract

The spatial organization of the genome plays a critical role in regulating gene expression. Recent chromatin interaction mapping studies have revealed that topologically associating domains and subdomains are fundamental building blocks of the three-dimensional genome. Identifying such hierarchical structures is a critical step toward understanding the three-dimensional structure–function relationship of the genome. Existing computational algorithms lack statistical assessment of domain predictions and are computationally inefficient for high-resolution Hi-C data. We introduce the Gaussian Mixture model And Proportion test (GMAP) algorithm to address the above-mentioned challenges. Using simulated and experimental Hi-C data, we show that domains identified by GMAP are more consistent with multiple lines of supporting evidence than three state-of-the-art methods. Application of GMAP to normal and cancer cells reveals several unique features of subdomain boundary as compared to domain boundary, including its higher dynamics across cell types and enrichment for somatic mutations in cancer.

## Abstract (web summary)

Spatial organization of the genome plays a crucial role in regulating gene expression. Here the authors introduce GMAP, the Gaussian Mixture model And Proportion test, to identify topologically associating domains and subdomains in Hi-C data.

## Introduction

Recent chromatin interaction mapping studies have revealed that mammalian genomes are organized hierarchically into domains of various sizes^1–4^. In particular, megabase-sized topologically associating domain (TAD) appears to a fundamental building block of the three-dimensional genome. Within each TAD, there exist subdomains. For instance, promoters and enhancers tend to form ~100 kb co-regulated clusters^2^. Identifying chromatin domains such as TADs and subTADs in different cell types is a critical step toward understanding the three-dimensional structure–function relationship of the genome. Several computational methods for identifying TADs^1, 4–8^ have been reported. Dixon et al.^1^ proposed a Hidden Markov Model for identifying TADs based on the directionality index, which quantifies the degree of upstream or downstream chromatin interaction bias at the periphery of the topological domains. Like the Dixon et al.^1^ method, Fraser et al.^9^ also used the directionality index but a different procedure to identify TADs. Flippova et al.^5^ proposed the *Armatus* algorithm, which is able to predict domains across various resolutions. Levy-Leduc et al.^10^ developed a two-dimensional-segmentation-based algorithm, HiCseg. The Arrowhead algorithm proposed by Rao et al.^4^ is computationally efficient with dynamic programming. Although these pioneering methods provide effective tools for TAD calling, several significant issues remain to be addressed. First, there is a lack of statistical significance assessment of predicted TADs. Second, previous methods also lack a principled strategy of choosing algorithmic parameters. For example, Dixon’s algorithm uses a prefixed window size of 2 Mb during TAD search. The arrowhead algorithm uses a heuristic strategy of tuning parameters. Finally, most existing methods cannot predict hierarchical domain structures. Weinreb and Raphael^7^ introduced the first algorithm for identifying hierarchical domains, TADtree, which can detect TADs and subTADs simultaneously by optimizing an objective function that scores a hierarchy of nested TADs. However, TADtree is rather slow with a running time of *O*(*S* ^5^) where *S* is the maximum TAD size.

In this paper, we describe the Gaussian Mixture model And Proportion test (GMAP) algorithm for identifying TADs and subTADs. GMAP specifically addresses the following issues, including treatment of noise in the chromatin interaction data, choice of optimal parameters of the method, and statistical significance of domain boundaries. Using both simulated and multiple types of experimental data, we demonstrate that GMAP achieved significantly improved accuracy and running speed. We applied GMAP to Hi-C data for multiple normal and tumour cell types to gain insights into the dynamics of subTADs and the relationship between domain boundary, somatic mutations, and enhancer-promoter communication in cancer.

## Results

### GMAP algorithm

**Fig. 1:**
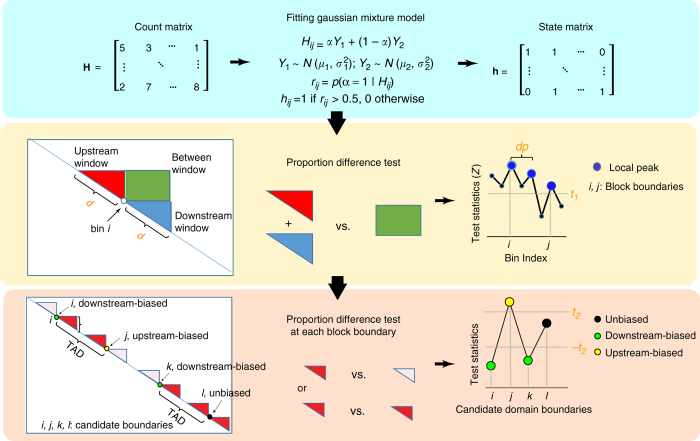
Overview of the GMAP method. The method consists of three major steps. In step one, we fit a Gaussian mixture model with two components representing chromatin interactions within and outside of a domain. In step two, for each genomic bin, we determine if it is located at the boundary of blocks of dense chromatin interactions by performing a proportion test of observed contact counts within and between windows flanking the bin. In step three, we call chromatin domains based on the location and orientation of the candidate boundaries identified in step two

The algorithm consists of three major steps (Fig. 1). The input to the algorithm is a normalized Hi-C contact matrix, *H*. In the first step, by fitting a two-component Gaussian mixture model to the normalized Hi-C count matrix, we distinguish contacts that are within a chromatin domain (intra-domain contacts) from contacts that are outside of a chromatin domain (*h*_*ij*_) (inter-domain contacts). This procedure also serves to further reduce the noise in the normalized Hi-C data. In step two, the algorithm uses a moving bin to scan along a chromosome. At each bin, the algorithm performs a proportion test (test statistic *Z*_*i*_), comparing intra-domain contact count of windows up- and down-stream of the bin to that of between up- and down-stream windows. The set of bins associating with the local peaks of test statistics (filtered by significant *P*-value) are called block boundaries and serves to partition a chromosome into blocks of dense chromatin interactions and gaps. In the third step, we define another test statistic, *D*_*i*_, to indicate whether a block boundary is upstream-biased, downstream-biased, or unbiased. Based on the relative orientation of its boundary, a block from step 2 can be called as a TAD, or merged into a larger TAD, or called as a gap between TADs. Once a TAD is called, we apply the same three steps to the normalized Hi-C data to call its subTADs until no element of the test statistic {*Z*_*i*_}_{1≤*i*≤*n*}_ is significant and/or the domain size is smaller than a pre-specified value.

To identify optimal parameter values of the algorithm, we introduce an objective function that maximizes the difference in the proportion of intra-domain contacts in putative domains and outside of putative domains (see Methods for details, Supplementary Table 3).

### Performance comparison using simulated Hi-C data

**Fig. 2:**
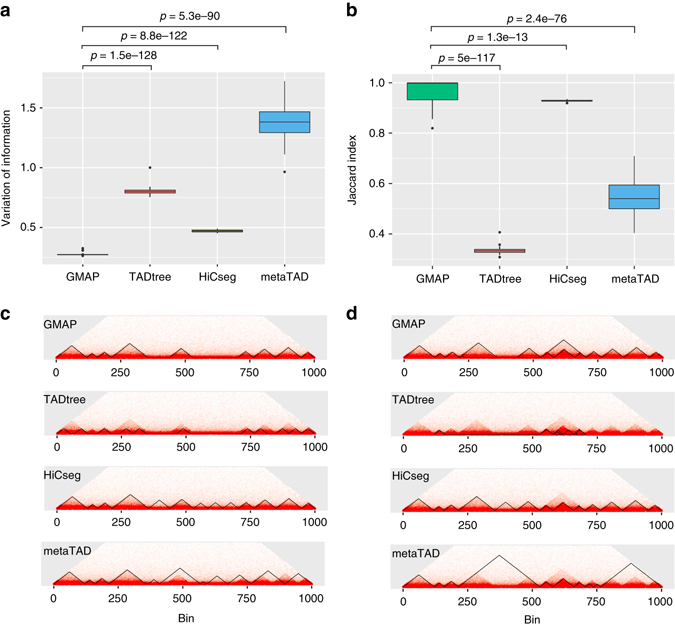
Performance comparison using simulated data. Hi-C contact count matrices were simulated using Poisson distribution. **a** Overall similarity between predicted and true domains measured using the Variation of Information (*VI*) index. **b** Overall similarity between predicted and true domains measured using the Jaccard Index. Shown are *boxplots* of VI and Jaccard indices over 100 simulations. The whiskers represent the most extreme data point which is no more than 1.5 times the interquartile range. Paired *t*-test was used to compare the performance metrics (VI or Jaccard index) for different methods. *P*-values are based on paired *t*-test. **c** An example of called TADs by different methods using simulated Hi-C data without embedded sub-TADs. Called domains are outlined by *solid black lines*. **d** An example of called TADs by different methods using simulated Hi-C data with embedded TADs and sub-TADS

We first used simulated Hi-C data to compare the performance of GMAP to three recently published methods, TADtree, HiCseg^10^, and metaTAD^9^. TADtree uses dynamic programming to detect a hierarchy of nested TADs and subTADs. HiCseg uses a maximum likelihood approach to partition Hi-C data into TADs. metaTAD first identifies TADs based on directionality index. It then combines TADs into larger domains called metaTADs. In this study, we only compared metaTAD with GMAP at the TAD level. Using Poisson distribution, we simulated two types of Hi-C contact matrices with a size of 1000 × 1000 bins. The first type only contains non-overlapping TADs, whereas the second type contains both TADs and subTADs. For both types of simulations, we took into account the effect of genomic distance on contact frequency and size distribution of TADs and subTADs in published literature (see Methods for details). We used two similarity measures to quantify the agreement between predicted and true domains, Variation of Information (VI) and Jaccard Index. A small value of VI and large value of Jaccard Index suggest better agreement between two partitions of a set. To evaluate performance variation due to statistical variation in simulated data, we generated 100 sets of simulated matrices and computed the similarity measures over the 100 sets of matrices. As shown in Fig. 2a, TADs called by GMAP have significantly higher agreement with the true TADs compared to TADs called by the other three methods. Figure 2c shows an example of predicted TADs by the four methods.

In terms of subTAD identification, GMAP correctly identified all subTADs (Fig. 2b, d). The other three methods correctly identified several subTADs but also produced several false positive subTADs and missed several TADs. Considering both TADs and subTADs, GMAP has the best overall accuracy (Fig. 2a, b). We also simulated Hi-C matrices using negative binomial distribution and found that GMAP outperformed the other three methods (Supplementary Fig. 1).

### Performance comparison using experimental Hi-C data

**Fig. 3:**
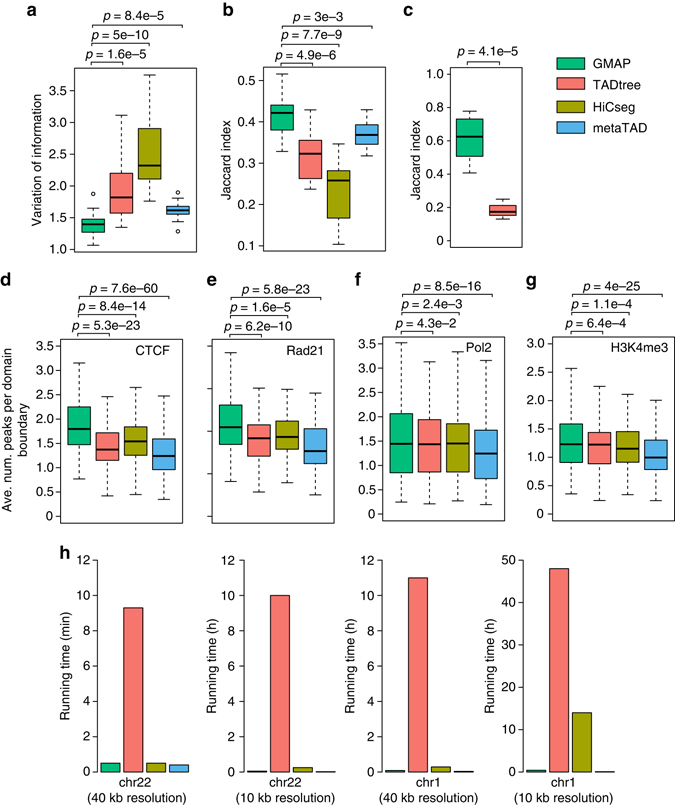
Performance comparison using experimental Hi-C data**. a**, **b** Similarity between TADs called using *low-resolution* (10 kb) and *high-resolution* (10 kb) Hi-C data. Hi-C data for the human lung fibroblast cell line, IMR90, was obtained from refs ^1, 4^. Similarity was measured using Variation of Information (*left*) and Jaccard Index (*right*). **c** Similarity between subTADs called using Hi-C and 5 C data. No data is shown for HiCseg and metaTAD since they do not call subTADs. Average number of CTCF peaks **d**, Rad21 peaks **e**, Pol2 peaks **f**, and H3K4me3 peaks **g** per TAD boundary. Values represent the average number of peaks within a TAD boundary plus 25 kb flanking regions on either side of the boundary across all chromosomes and six cell types (IMR90, GM12878, NHEK, HMEC, HUVEC, and K562). *P*-values are based on paired *t*-test. The whiskers represent the most extreme data point which is no more than 1.5 times the interquartile range. **h** Running speed of different methods

Due to the scarcity of experimentally validated chromatin domains, we resorted to the following three strategies to further evaluate the quality of predicted TADs and subTADs: (1) agreement of domains predicted for the same cell type using Hi-C data with different resolutions; (2) agreement of domains predicted for the same cell type but using data generated with different technologies (i.e., Hi-C vs. 5C); (3) enrichment of known domain boundary factors at predicted domain boundaries. A good method should produce similar results when using Hi-C data generated at different resolutions. We applied the four methods to the normalized Hi-C data for the human lung fibroblast cell line, IMR90, at both low-resolution (40 kb) and high-resolution (10 kb), downloaded from ref. ^1^ and ref. ^4^, respectively (Supplementary Table 1). Two examples of TADs and subTADs called by GMAP are shown in Supplementary Fig. 2. We then examined the similarity between the two sets of TADs called by the same method. We found that TADs called by GMAP at both resolutions have significantly higher similarity than TADs called by the other three methods, indicating that GMAP generates more consistent results across different resolutions (Figs. 3a, b).

We further compared subTADs called using 5C data and Hi-C data for the same cell type. In general, 5C data has higher resolution than Hi-C data and thus better suited for identifying subTADs. Based on this observation, we reasoned that similarity among subTADs called by the same method using different data types (Hi-C vs. 5C data) can serve as a performance measure. To this end, we used a high-resolution 5C data set and a Hi-C data set for mouse embryonic stem cells^11^. The 5C data was preprocessed and normalized using the HiFive^12^ tool. As shown in Fig. 3c, the two sets of subTADs called by GMAP are significantly more similar than those called by TADtree (*P* = 4.1E-5, *t*-test). HiCSeg and metaTAD were not compared since they cannot predict subTADs.

It has been reported that chromatin domain boundaries are frequently occupied by several protein and epigenetic factors, including the genome architectural protein CTCF, cohesion complex, promoters of highly transcribed genes, and the histone mark H3K4me3. We examined the enrichment of these known factors at predicted domain boundaries. Specifically, for all four methods, a domain boundary is represented by a bin and thus has the same size. We examined the presence of factor peaks in the region that span the domain boundary and the 25 kb region flanking the boundary. In all six human cell types examined (IMR90, NHEK, GM12878, HMEC, HUVEC, and K562, Supplementary Tables 1 and 2), we found that TADs boundaries reported by GMAP are significantly more enriched for the known factors than boundaries reported by TADtree, HiCseg, and metaTAD (Fig. 3d–g).

Domain identification is computationally intensive given the size of a typical Hi-C contact matrix (~25 million cells at 40 kb resolution). Such matrices will become much larger with the increasing resolution of Hi-C data. We thus evaluated the running speed of the three methods. As shown in Fig. 3h, GMAP has comparable speed as HiCseg and metaTAD using data from a small chromosome (Chr 22, 51 Mb) at both low and high resolutions (*left* two panels). As the data size becomes much bigger (Chr1, 249 Mb, comparing *left* two panels to *right* two panels), the speed advantage of GMAP over TADtree and HiCseg becomes more dramatic.

In summary, using multiple data types, we demonstrated that GMAP achieved significant improved accuracy and running speed.

### SubTADs are more dynamic than TADs across cell types

**Fig. 4:**
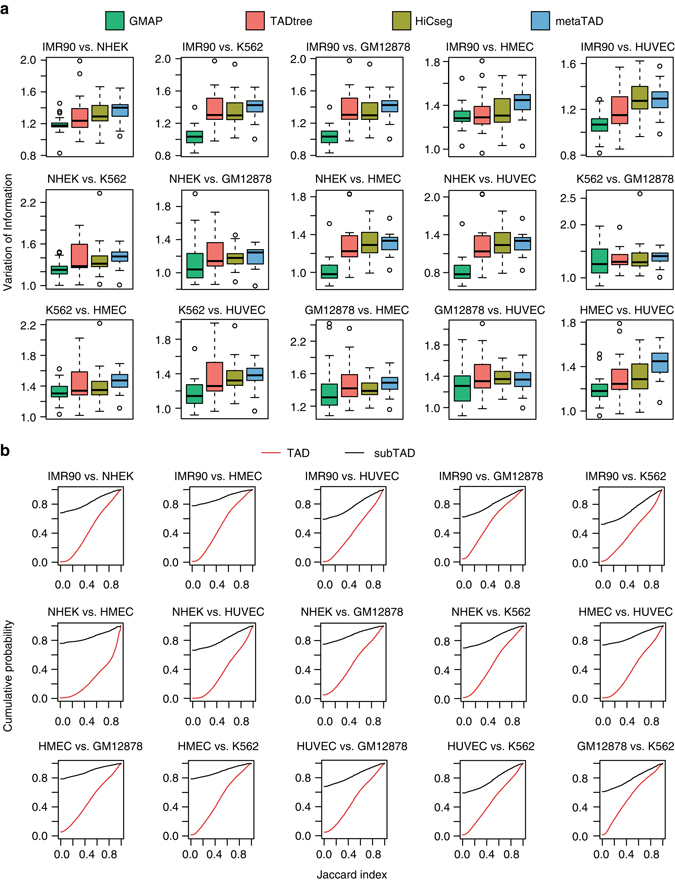
SubTAD boundaries are more dynamic than TAD boundaries across different cell lines. **a** Pairwise similarity of TADs across six human cell lines, GM12878, HMEC, HUVEC, IMR90, K562, NHEK. In all but four pairwise comparisons, the difference between GMAP and the other three methods is significantly different based on *t*-test (*P* < 0.05). The whiskers represent the most extreme data point which is no more than 1.5 times the interquartile range. **b** Comparison of pairwise similarities for TADs and subTADs across six human cell lines. The cumulative probability plots show that subTAD boundaries are more dynamic across all pairwise comparisons of the six cell lines. In all pairwise comparisons, the cumulative distribution for subTADs is significantly different than the distribution for TADs based on KS test (*P* < 0.05)

An important observation from all Hi-C studies so far is that TAD boundaries do not vary significantly across different cell types^13^. To further evaluate the performance of GMAP, we performed a systematic analysis of TADs using high-resolution Hi-C data from six human cell lines and three TAD callers. In all pairwise comparisons, we found that TADs between two cell types identified by GMAP are more similar than TADs identified by the other two methods (Fig. 4a). Thus, prediction by GMAP is more consistent with previous conclusion that TAD boundary is fairly static. Beyond TADs, given the hierarchical nature of genome organization, it is important to understand the dynamics of chromatin domains at lower hierarchy. For instance, within TADs, promoters and enhancers have been found to form ~100 kb co-regulated clusters^2^. It was also reported that HoxA genes and their regulatory elements physically interact with each other through contacts between subTADs^14^. Taken together, these earlier studies suggest that subdomains play an important role in gene regulation. So far, no systemic comparison has been done regarding subTAD boundaries. By comparing subTADs predicted by GMAP across the six cell types, we found that subTAD boundaries are consistently more dynamic than TAD boundaries (Fig. 4b), suggesting different molecular mechanisms may be responsible for the formation of TADs and subTADs.

To further examine domain dynamics during development, we analyzed Hi-C data during mouse embryonic stem cell differentiation to neuron via neural progenitor cells^9^. We again found that subTAD boundaries are consistently more dynamic than TAD boundaries during the differentiation process (Supplementary Fig. 3). Taken together, our analysis suggests that subTADs are more dynamic during development and across different cell types.

### Relationship between domain organization and cancer mutation

**Fig. 5:**
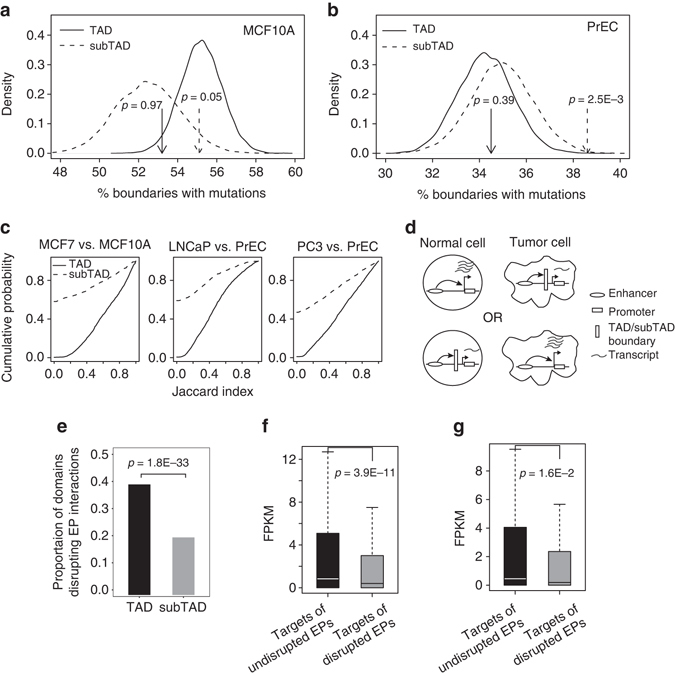
Relationship between hierarchical domain boundary, somatic mutation, and enhancer–promoter communication**. a**, **b** SubTAD but not TAD boundaries are enriched for somatic mutations in cancer. Percentage of TAD and subTAD boundaries overlapping with at least one recurrent mutations for MCF10A cells **a** and PrEC cells **b**. TAD and subTADs were identified using Hi-C data for non-tumorigenic mammary gland epithelial cell line (MCF10A) and prostate epithelial cell line (PrEC). *Solid line*, TADs; *Dashed line*: subTADs. Observed percentages are indicated by *vertical lines* with an *arrow*. Distributions of expected percentages are generated using 10,000 sets of randomly selected genomic regions with the same number and size as the called TADs/subTADs for each cancer cell type. **c** SubTAD boundaries are more dynamics than TAD boundaries between cancer and normal cells. MCF7, breast adenocarcinoma cell line; LNCaP, prostate carcinoma cell line; PC3, prostate adenocarcinoma cell line. **d** Schematic demonstrating that cell-type-specific enhancer–promoter (*EP*) interactions are blocked by newly formed domain boundary in the other cell type. **e** Proportion of cell-type-specific domain boundaries that overlap with cell-type-specific EP interactions in the other cell type. *P*-value is based on *t*-test. **f** Expression levels of promoters involved in cell-specific EP interactions that are blocked by TAD boundary in the other cell type. **g** Expression levels of promoters involved in cell-specific EP interactions that are blocked by subTAD boundary in the other cell type. *P*-values are based on *t*-test. The whiskers represent the most extreme data point which is no more than 1.5 times the interquartile range

Previous studies have demonstrated that disruption of TAD boundaries can result in deregulated gene expression and disease^15^. Furthermore, boundaries of insulated chromatin neighborhoods (defined as regions enclosed by a pair of genomic sites co-occupied by CTCF and cohesin) are enriched for somatic mutations in cancer^16^. The fact that TAD and insulated chromatin neighborhood have very different sizes suggests that mutations can affect domain boundaries at different hierarchies. To better understand the relationship between hierarchical domain boundaries and genetic mutation, we examined the frequency of somatic mutations at both TAD and subTAD boundaries. We downloaded recurrent somatic mutations identified using whole-genome sequencing by the International Cancer Genome Consortium (ICGC) (Supplementary Table 4). We then applied GMAP to Hi-C data for both benign (MCF10A and PrEC) and malignant (MCF7, LNCaP, PC3) breast cancer^17^ and prostate cancer cell lines^18^. Interestingly, for both cancer types, we found that subTAD boundaries in both normal and tumor cells are significantly enriched for somatic mutations while TAD boundaries are not enriched (Fig. 5a, b and Supplementary Fig. 4). This result suggests that subTAD boundaries are more susceptible to genetic mutations in cancer. Consistent with our analysis with six cell lines in previous section, we also observed that subTADs are more dynamic between benign and tumor cells than TADs (*P* < 0.05, KS test, Fig. 5c). This higher level of dynamics of subTADs may be linked to the higher frequency of somatic mutations at their boundaries.

### Cancer-specific domain boundary blocks enhancer–promoter (EP) interactions

To further understand the impact of domain boundary re-organization on EP interaction and gene expression in cancer, we intersected domain boundary calls with EP interactions predicted using the IM-PET algorithm^19^. We considered the situation where a cell-type-specific EP interaction (e.g., normal cell) spans a boundary region that is only observed in the other cell type (e.g., cancer cell) and vice versa (Fig. 5d), which suggests that the formation of cell-type-specific boundary disrupts the EP interaction in the former cell type. Compared to subTAD boundaries, we found a significantly higher proportion of TAD boundaries whose formation block EP interactions in the other cell type (Fig. 5e). This is consistent with the finding that TAD are more stable than subTAD^13^, which in turn suggests that ectopically formed TAD boundary are more likely to disrupt EP interactions. To further examine the impact of the disrupted EP interactions, we compared the expression levels of involved promoters in normal and cancer cells or vice versa. We found that promoters of disrupted EP interactions have significantly lower expression in the cell type in which the EPs are disrupted due to boundary formation. This is true for both TAD and subTAD boundaries (Fig. 5f, g). Interestingly, the significance of expression decrease due to TAD boundary formation is nine orders of magnitude higher than the significance of expression decrease due to subTAD boundary formation (3.9E-11 vs. 1.6E-2, *t*-test), further supporting the notion that TAD boundary is more rigid and once formed is more effective in blocking EP interactions and disrupting gene expression.

## Discussion

There is a critical need for algorithms to analyze Hi-C data given the latest explosion of such data type. Of particular interest are algorithms for simultaneous identification of chromatin domains at multiple organizational hierarchies. Several algorithms have been reported for detecting TADs^1, 4, 5, 10^, but few for detecting hierarchical chromatin domains. We introduce the GMAP algorithm, for detecting hierarchical chromatin domains from Hi-C data. GMAP addresses several deficiencies of existing algorithms. First, most domain callers donot explicitly consider noise in the contact count matrix caused by random chromatin looping. By using Gaussian mixture modeling, GMAP distinguishes intra-domain contacts from inter-domain contacts. An additional advantage of Gaussian mixture models is their flexibility of modeling a wide range of probability distributions. In contrast, previous methods, such as HiCseg has a strong assumption on the form of distribution for Hi-C count data. Second, GMAP includes a statistical test to assess the significance of putative domain boundaries. Finally, GMAP substantially improves upon the running speed of TADtree, the only published algorithm for detecting subTADs.

There are a number of ways GMAP can be improved. First, we use an iterative procedure to identify subTADs. Thus, the accuracy of subTADs depend on the accuracy of the enclosing TADs. Novel strategy is needed for reducing such dependency in order to increase the accuracy of subTADs. Second, although GMAP is faster than existing algorithms, it may not be enough for the fast increasing amount of Hi-C data. The speed of GMAP can be further improved by using parallel computing framework and graphics processing unit. Third, other types of omics data, such as epigenomic data and transcriptomic data should be combined with Hi-C to further improve the accuracy of hierarchical domain calling.

Previous studies mostly focus on TADs. Here, application of GMAP to Hi-C data from multiple cell types has revealed some unique features about subTADs, including higher dynamics (among different cell types as well as between benign and tumour cells) and higher proportion of somatic mutations at subTAD boundary. Such features warranty future experimental studies to better understand the impact of hierarchical chromatin organization on a number of genome transactions, such as replication, transcription, and mutation.

## Methods

### Major steps of the GMAP algorithm

In step one of the algorithm, we model the normalized Hi-C data matrix by a two-component Gaussian mixture model (Fig. 1a). Our rationale is that observed chromatin contacts can be categorized into two types: “intra-domain contact” and “inter-domain contact”. We denote the normalized Hi-C data matrix as **H**, with component *H*_*ij*_ representing the contact frequency between bins *i* and *j*. We focus on the upper triangle of **H** because it is symmetric. Let *Y*_1_ and *Y*_2_ denote the random variables for the observed “intra-domain contact” frequency and “inter-domain contact” frequency, respectively. The two-component Gaussian mixture model can be specified as:

$$\begin{array}{l} {Y_1} \sim N\left( {{\mu _1},\sigma _1^2} \right),\\ {Y_2} \sim N\left( {{\mu _2},\sigma _2^2} \right),\\ {H_{i,j}} = \alpha {Y_1} + \left( {1 - \alpha } \right){Y_2}\\ \end{array}$$

where N(*μ*, *σ* ^2^) represents a Gaussian distribution with mean *μ* and variance *σ*,^2^ respectively and *α* is the mixing coefficient. The model parameters *μ*_1,_ *μ*_2_, $\sigma _1^2,\sigma _2^2$ can be estimated using the Expectation-Maximization algorithm. To distinguish intra-domain from inter-domain contacts, we use the posterior probability, $\widehat {{r_{i,j}}} = {\rm{Pr}}\left( {\alpha = 1|{H_{i,j}}} \right)$. The Hi-C count matrix is transformed into a state matrix using the following criterion on $\widehat {{r_{i,j}}}$:

$${h_{i,j}} = \left\{ {\begin{array}{*{20}{c}} {1\,if\widehat {{r_{i,j}}}  >0.5} \\ {0\, {\rm {otherwise}}} \\ \end{array}} \right. \cdot $$

In the second step of the algorithm, we perform a proportion test to identify blocks of dense chromatin interactions (Fig. 1b). We use a moving bin to segment a chromosome into blocks of dense chromatin interactions and gaps (unstructured regions between domains). For each genomic bin, we first define its upstream window and downstream window, and compare the proportion of intra-domain contacts within both windows to the proportion of intra-domain contacts between the windows. Specifically, for bin *i* we define its upstream window as the union of bins between bin *i*−*d*+1 and bin *i* and its downstream window as the union of bins between bin *i* and bin *i*+*d*−1, where *d* is a parameter of the method. Let $p_i^w$and $p_i^b$be the above-mentioned proportions within and between windows, and

$$p_i^w = \frac{{{{\mathop {\sum}\nolimits_{i - d + 1 \le j,k \le i} h }_{jk}} + {{\mathop {\sum}\nolimits_{i \le j,k \le i + d - 1} h }_{jk}}}}{{2{d^2}}}$$

$$p_i^b = \frac{{{{\mathop {\sum}\nolimits_{i - d + 1 \le j \le i,i \le k \le i + d - 1} h }_{jk}}}}{{{d^2}}}.$$

We then construct a test statistic ${Z_i} = \left( {p_i^w - p_i^b} \right)/\sqrt {{p_{0i}}\left( {1 - {p_{0i}}} \right)/{d^2}}$ with ${p_{0i}} = \left( {p_i^w + p_i^b} \right)/2$. Denote the sequence of local test statistics peaks as ${\left\{ {{{\bf{Z}}_i}} \right\}_{\left\{ {1 \le i \le n} \right\}}}$ (or equivalently the corresponding *P*-value) that are located at positions *l*_1,_ *l*_2,_…,*l*_*k*_. They are used to segment the chromosome into blocks of dense interactions and gaps. We use a threshold *t*_1_ to call local peaks; if ${Z_{{l_i}}} \le {t_1}$, it is not recognized as a local peak, thus *t*_1_ can be used to control the size of the block. We also combine local peaks that are very close; given parameter *d*_*p*_, for peak positions satisfying $\left| {{l_i} - {l_{i + 1}}} \right| \le {d_p}$, remove *l*_*i*_ from the sequence of peaks if ${Z_{{l_i}}} \le {Z_{{l_{i + 1}}}}$and remove *l*_*i*+1_ otherwise. *d*_*p*_ controls the minimal size of a chromatin domain.

In the third step of the algorithm, we perform domain calling by combining block boundaries (Fig. 1c). A block from step 2 can be called as a TAD, or merged into a larger TAD, or called as a gap between TADs. To do this, we define another test statistic to indicate whether a bin is upstream-biased, downstream-biased or unbiased. Let $p_i^u$ and $p_i^d$ denote the proportion of real contacts in upstream and downstream windows, respectively, and

$$p_i^u = \frac{{\mathop {\sum }\nolimits_{i - d + 1 \le j,k \le i} {h_{jk}}}}{{{d^2}}},p_i^d = \frac{{\mathop {\sum }\nolimits_{i \le j,k \le i + d - 1} {h_{jk}}}}{{{d^2}}}.$$

The test statistic is defined as ${D_i} = \left( {p_i^u - p_i^d} \right)/\sqrt {{p_i}\left( {1 - {p_i}} \right)/{d^2}}$ with ${p_i} = \left( {p_i^u + p_i^d} \right)/2$. We determine the *ith* bin’s directionality as upstream-biased if *D*_*i*_ > *t*_2_, downstream-biased if *D*_*i*_ < −*t*_2_ and unbiased otherwise. A TAD starts from a downstream-biased peak and can continue to include several consecutive downstream-biased peaks. A TAD ends when an upstream-biased peak or unbiased peak is reached. An unbiased peak is shared by two consecutive TADs. Chromosomal regions start from an up-biased peak, extend to a downstream-biased peak is called as gaps between two TADs.

In the above TAD calling procedure, parameter *t*_2_ is essential for deciding whether a block is a gap, or whether some consecutive blocks should be merged into a larger TAD.

### Tuning the parameters of the algorithm

There are four parameters *d*, *d*_*p*_, *t*_1_, and *t*_2_ in our method. We optimize them by maximizing the difference in the proportion of intra-domain contacts in TADs and non-TADs (background), which is defined as

$$\frac{{{p_{{\rm{TAD}}}} - {p_{bg}}}}{{\sqrt {p\left( {1 - p} \right)\left( {1/{n_{{\rm{TAD}}}} - 1/{n_{bg}}} \right)} }}$$

where ${p_{{\rm{TAD}}}} = \mathop {\sum}\nolimits_{(i,j) \in {\rm{TAD}}} {{h_{ij}}/{n_{{\rm{TAD}}}}}$, ${p_{bg}}=\mathop {\sum}\nolimits_{|i - j| \le D} {{h_{ij}}/{n_{bg}}}$, *p*=(*p*_TAD_ *n*_TAD_−*p*_*bg*_ *n*_*bg*_)/(*n*_*TAD*_+*n*_*bg*_) and *n*_TAD_ and *n*_*bg*_ are the total number of bin pairs within TADs and the background, respectively. Note that we define background as those bin pairs whose distance is less than or equal to a predefined number *D* and are not in the predicted TADs, which we set to 2 Mb based on the size of the largest TAD in published studies^1^. The ranges of parameter values searched in this study are provided in Supplementary Table 3.

### Iterative procedure for identifying sub-domains

Given a called TAD, we apply the GMAP algorithm again on the normalized Hi-C data to call its subTADs until no element of the test statistic {**Z**_*i*_}_{1≤*i*≤n}_ is significant and/or the domain size is smaller than a pre-specified value. In this report, we used 200 kb as the minimal domain size based on published studies^1^. We use the TAD itself as background when calling its subTADs.

### Simulation of Hi-C data

We generate contact count matrices with a size of 1000 × 1000 bins. Values in the contact matrix follow either a Poisson distribution or a negative binomial distribution. To account for the effect of distance on contact count due to random polymer interaction, we make the mean of the distribution proportional to the inverse of the distance between two bins, *i and j*, as defined in the following equation:

$${H_{ij}} = {\rm{Poisson}}\left( {\frac{u}{{\left| {i - j} \right|}}} \right),1 \le i,j \le 1000$$

Based on this equation, the average contact count in a smaller TAD is larger than that in a bigger TAD. TADs of varying sizes are inserted along the diagonal of the contact matrices. Specifically, for a TAD with size of *l* − *k*+1, we replace the sub-matrix of **H** corresponding to TAD by a matrix **T** as

$${T_{ij}} = {\rm{Poisson}}\left( {\frac{{t*u}}{{\left| {i - j} \right|}}} \right),l \le i,j \le k$$

where *t* represents the signal ratio of TAD over the background. We set it to 2 in the simulation study and *μ* is set to 200 to generate **H** with a mean value about 6, which is close to the mean contact count of real Hi-C matrix from Rao et al.^4^ (2014) at 40 kb resolution. We randomly select ten bins among the 1000 bins. We then embed ten TADs with sizes ranging from 40 to 175 bins. These simulated TAD sizes follow the size distribution of TADs reported in the literature. We also randomly embed two regions as gaps between TADs.

For sub-TADs, we use the same simulation strategy as for TADs. To insert subTADs, we replace the sub-matrix of **H** corresponding to the subTAD from bin *m* to bin *n* as

$${S_{ij}} = {\rm{Poisson}}\left( {\frac{{s*t*u}}{{\left| {i - j} \right|}}} \right),m \le i,j \le n$$

where *s* represents the signal ratio of subTAD over TAD and we set it to 2 as well.

Using the same strategy, we simulated Hi-C count matrix using negative binomial distribution. The mean parameters are set to the same values as in the Poisson distribution simulation, and then we choose the dispersion parameter such that the variance equals to 1.25 times the mean parameter for **H**, **T**, and **S**, respectively.

### Assessing agreement between two sets of chromatin domains

To compare two sets of domain calls, we used two metrics: VI and Jaccard Index. VI was defined for evaluating similarity between two partitions of a given set^20^. Given two partitions A and B of a set S into disjoint subsets, **A**={*A*_1_,…,*A*_*k*_}, **B**={*B*_1_,…,*B*_*l*_}, where *k* and *l* are the total number of subsets in A and B, respectively Let $n = \mathop {\sum}\nolimits_i {\left| {{A_i}} \right|} = \mathop {\sum}\nolimits_j {\left| {{B_j}} \right|} = \left| S \right|$, ${p_i} = \left| {{A_i}} \right|/n$, ${q_i} = \left| {{B_j}} \right|/n$, ${r_{ij}} = \left| {{A_i} \cap {B_j}} \right|/n$, where $\left| {{A_i}} \right|$ represents the size of subset *A*_*i*_. VI between the two partitions is:

$$VI\left( {A;B} \right) = - \mathop {\sum}\limits_{i,j} {{r_{ij}}} \left[ {\log \left( {{r_{ij}}/{p_i}} \right) + \log \left( {{r_{ij}}/{q_j}} \right)} \right].$$

We used VI to assess the agreement of TADs identified from two data sets by the same method, or from the same data set but by different methods. Note that since VI requires the subsets in a partition to be disjoint, it cannot be used to evaluate hierarchical partitions involving subTADs. To address this issue, we used Jaccard Index. Given the above-mentioned set notations, the Jaccard Index of any pair of domains can be defined as

$${\rm{Jaccard}}\left( {{A_i},{B_j}} \right) = \frac{{\left| {{A_i} \cap {B_j}} \right|}}{{\left| {{A_i} \cup {B_j}} \right|}}$$

The Jaccard Index of *A*_*i*_ to the partition set *B*, which quantifies the best match of *B* to *A*_*i*_, can be defined as

$${\rm{Jaccard}}\left( {{A_i},B} \right) = {{\rm{max}}_{1 \le j \le l}}{\rm{Jaccard}}\left( {{A_i},{B_j}} \right)$$

The Jaccard Index of *B*_*J*_ to set *A* is defined similarly.

Domain coordinates are represented either as bin indices (simulated data) or real chromosome coordinates. No threshold is used to measure overlap of two sets of domains because both VI and Jaccard Index are threshold independent.

### Hi-C data processing and normalization

Hi-C data for cancer cell lines (K562, MCF7, LNCaP, PC3) were normalized using the algorithm caICB, which is designed for correcting biases due to copy number variation in cancer genomes^21^. Hi-C data for non-cancer cell lines (MCF10, PrEC) were processed and normalized using HiC-Pro^22^. The normalized Hi-C data from Dixon et al.^1^ and Rao et al.^4^ were downloaded from GEO using accession numbers provided by the authors.

### Parameter setting for compared methods

For simulation studies using TADtree, we set the number of outputted TADs as the average of the number of true TADs and the number of TADs outputted by HicSeg. The parameter *M* (maximal number of subTADs within TADs) were set to 3. The other parameters of TADtree were set as the default values. For HiCseg using simulated data, although it has three modeling options using Gaussian, or Poisson or Negative Binomial distribution, we used Poisson distribution for the simulated data set because we found that negative binomial distribution did not always converge and Gaussian distribution is not appropriate for modeling count data. The other parameters of HiCseg were set as the default values except for parameter *Kmax* which was set to 80. For metaTAD, *L* was set as 50 bins (the same as the default 2 Mb if the resolution is 40 kb) and *α* = 0 as used in the original publication. For analyzing experimental Hi-C data with TADtree, we set the number of outputted TADs as the average of the number of true TADs and the number of TADs outputted by HicSeg. The other parameters were set as default values for low-resolution Hi-C data. Parameters *S* and *N* were set to 200 and 400 for high-resolution Hi-C data. For HiCseg, we use Gaussian distribution because we used normalized Hi-C data. The other parameters were set as the default values except for *Kmax*, which was set to 500 for high-resolution Hi-C data. For metaTAD, both *L* and α were set as default values as in the original publication.

### Enrichment analysis of genomic factors at domain boundaries

ChIP-Seq data was downloaded from Gene Expression Omnibus. Accession numbers are provided in Supplementary Table 2. We calculated the average numbers of CTCF, RAD21, H3K4me3, and Pol2 peaks within the 25 kb region flanking a TAD boundary on both side (and including the boundary). Statistical significance in the mean peak number within TAD boundaries was computed using paired *t*-test.

### RNA-Seq analysis

RNA-Seq data for normal and breast cancer cell lines were downloaded from ref. ^17^. Sequencing reads were mapped using TopHat^23^ (version 2.1.1). Cufflink tool^24^ was used to calculate expression level as fragments per kilobase of transcript per million reads (FPKM). The average FPKM across the three replicates was used for downstream analysis.

### Assumptions of statistical tests

All statistical tests were performed using large sample sizes. Other assumptions of specific tests such as normal distribution and equal variance for *t*-test were tested to be satisfied before conducting the real tests. Therefore, the test results are robust with regard to underlying assumptions of the statistical tests.

### Code availability

An R package *rGMAP* implementing the GMAP algorithm is available at the following website: http://tanlab4generegulation.org/rGMAP_1.1.tar.gz.

### Data availability

The data that support the findings of this study are available from the Gene Expression Omnibus (GEO) database. Accession numbers are listed in Supplementary Tables 1 and 2.

## Electronic supplementary material

Supplementary Information
