# Supplementary material for: Identifying topologically associating domains and subdomains by Gaussian Mixture model And Proportion test
Source: Nat Commun. 2017 Sep 14;8:535. doi: 10.1038/s41467-017-00478-8 (PMC5599511; doi:10.1038/s41467-017-00478-8)
Supplement: Supplementary file 1 — Supplementary Information [file 41467_2017_478_MOESM1_ESM.pdf]

Description of Supplementary Files

File name: Supplementary Information
Description: Supplementary figures, supplementary tables, supplementary methods and supplementary references.

Supplementary Methods

Fitting of Gaussian mixture models using Expectation-Maximization algorithm

We used the popular R package ‘mclust’ to implement the Expectation-Maximization (EM) algorithm. Within mclust, EM algorithm is initialized using the partitions obtained from model-based hierarchical agglomerative clustering (MBHAC). Although there is no guarantee that the EM initialized by MBHAC will converge to the global optimum, it often provides reasonable starting points (Scruca et al, The R Journal Vol. 8/1, 2016). As for model training, mclust trains the Gaussian mixture models with a variety of covariance structures and Bayesian Information Criterion (BIC) was used for selecting the final model.

Parameter searching

We provided the default parameter search intervals for each of the four parameters of GMAP algorithm in Supplemental Table 3. The optimized default values the parameters are recommended for calling domains using Hi-C data with 40kb and 10kb resolution. These values were motivated by the TAD sizes published in Dixon et al. (2012) and Rao et al. (2014). For Hi-C data with resolution higher than 10kb or lower than 40kb, users can increase or decrease the value for *d*.

Supplementary Table 1. Source of Hi-C data used in this study.

Cell line	Resolution	Normalization Method (reference)	Source or [Accession Number]
IMR90	40kb	(2)	(2)
IMR90	10kb	KR normalization (3)	[GSE63525]
GM12878	10kb	KR normalization (3)	[GSE63525]
NHEK	10kb	KR normalization (3)	[GSE63525]
K562	10kb	KR normalization (3)	[GSE63525]
HMEC	10kb	KR normalization (3)	[GSE63525]
HUVEC	10kb	KR normalization (3)	[GSE63525]
MCF-7	40kb	ICE (4)	[GSE66733]
MCF-10A	40kb	ICE (4)	[GSE66733]
LNCaP	40kb	ICE (4)	[GSE73785]
PC3	40kb	ICE (4)	[GSE73785]
PrEC	40kb	ICE (4)	[GSE73785]
ESC	40kb	ICE (4)	[GSE59027]
NPC	40kb	ICE (4)	[GSE59027]
Neuron	40kb	ICE (4)	[GSE59027]

Resolution represents the size of the genomic bin used for summing up Hi-C contact counts in the bin.

Supplementary Table 2. Source of ChIP-Seq data used in this study.

Factor	Cell line [accession number]	Notes
CTCF	IMR90 [GSM935404], K562 [GSM733719], GM12878 [GSM733752], HMEC [GSM733724], NHEK [GSM733636], HUVEC [GSM733716]	The ChIP-Seq peaks can be found in the published work corresponding to the above provided accession number.
H3K4me3	IMR90 [GSM469970], K562 [GSM733680], GM12878 [GSM733708], HMEC [GSM945159], NHEK [GSM733720], HUVEC [GSM733673]	
RAD21	IMR90 [GSM935624], K562 [GSM803447], GM12878 [GSM803416]	
POL2	IMR90 [GSM935513], K562 [GSM733643], GM12878 [GSM935608], NHEK [GSM733671], HUVEC [GSM733794]	

Supplementary Table 3. GMAP parameter values tested for calling TADs and subTADs using experimental Hi-C data.

Parameters	TADs	subTADs
$\boldsymbol{d}$	25, 50, 75, 100 bins	5, 10, …, half size of TAD bins
$\boldsymbol{d_p}$	5, 10 bins	5, 10 bins
$\boldsymbol{t_1}$	20 evenly distributed values between 50 and 95 percentile of the corresponding proportion test statistics	20 evenly distributed values between 50 and 95 percentiles of the corresponding proportion test statistics
$\boldsymbol{t_2}$	10 evenly distributed values between 50 and 95 percentiles of the corresponding proportion test statistics	10 evenly distributed values between 50 and 95 percentiles of the corresponding proportion test statistics

Supplementary Table 4. Summary of somatic mutation data obtained from International Cancer Genome Consortium (ICGC) data portal.

**Cancer Type**	**# simple somatic mutations (#. occurrence >= 3)**
Breast cancer	37,361
Prostate cancer	22,406

Here simple somatic mutations include point mutations, small insertions and deletions.

Supplementary Figure 1. Performance comparison using simulated data based on negative binomial distribution. A) Overall similarity between predicted and true domains measured using Variation of Information (VI) index. **B)** Overall similarity between predicted and true domains measured using Jaccard Index. Shown are boxplots of VI and Jaccard indices over 100 simulations. P-values are based on t-test. **C)** An example of called TADs by different methods using simulated Hi-C data without embedded sub-TADs. Called domains are outlined by solid black lines. **D)** An example of called TADs by different methods using simulated Hi-C data with embedded TADs and sub-TADS.

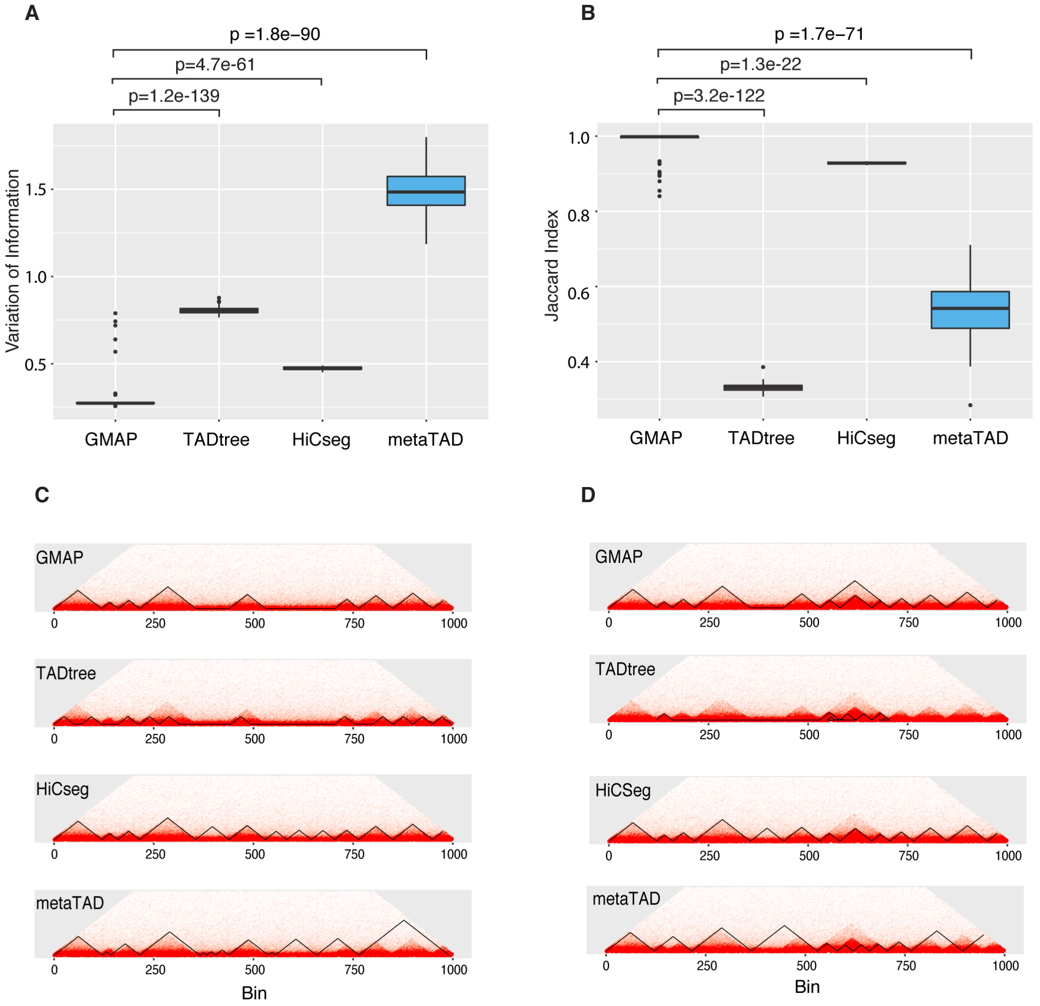

Supplementary Figure 2. Example TADs and subTADs in IMR90 cells called by GMAP. A) calls on chromosome 8. **B)** calls on chromosome 15.

A

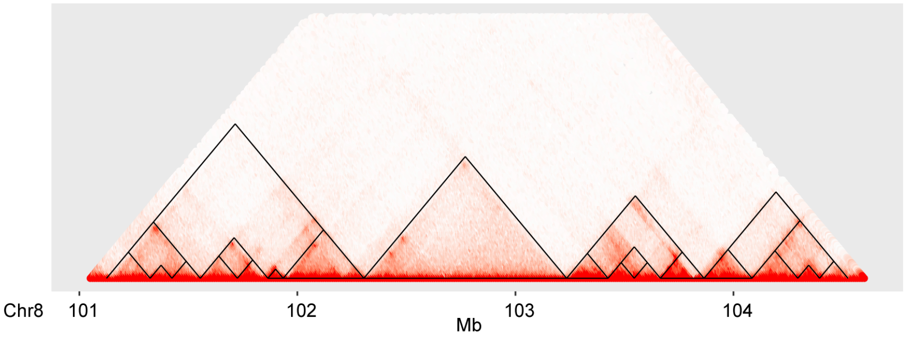

B

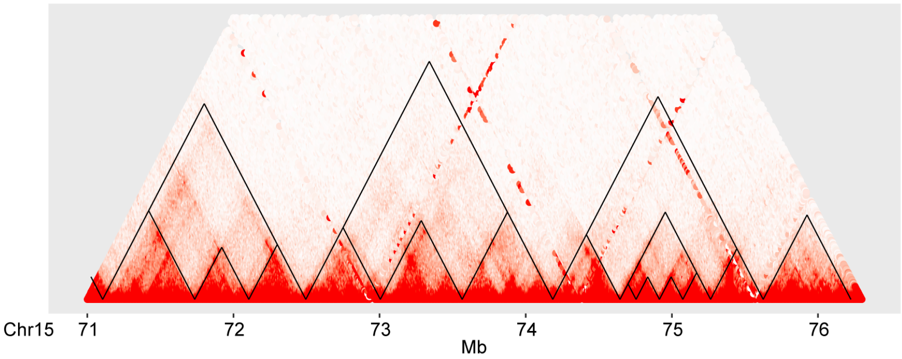

Supplementary Figure 3. Similarity of TADs and subTADs between two cell types during ESC differentiation. Pairwise similarities for TADs and subTADs across ESC, NPC, and neuron were compared. The cumulative probability plots show that subTAD boundaries are more dynamic across all pairwise comparisons of the three cell types. In all pairwise comparisons, the cumulative distribution for subTADs is significantly different than the distribution for TADs based on KS test (p<E-100).

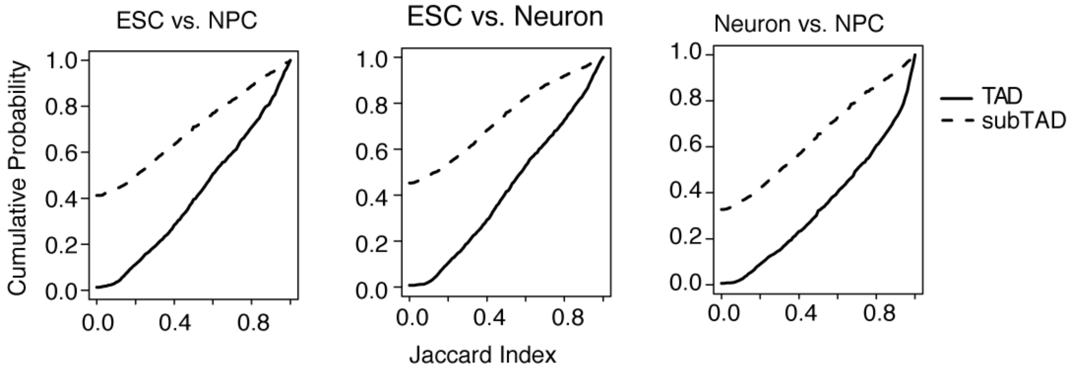

Supplementary Figure 4. **SubTAD but not TAD boundaries are enriched for somatic mutations in cancer.** Percentage of TAD and subTAD boundaries overlapping with at least one mutation for MCF7 cells **A)**, LNCaP cells **B)** and PC3 cells **C)**. TAD and subTADs were identified using Hi-C data for breast adenocarcinoma cell line (MCF7), prostate carcinoma cell line (LNCaP) and prostate adenocarcinoma cell line (PC3). Solid line, TADs; Dashed line: subTADs. Observed percentages are indicated by vertical lines with an arrow. Distributions of expected percentages are generated using 10,000 sets of randomly selected genomic regions with the same number and size as the called TADs/subTADs for each cancer cell type.

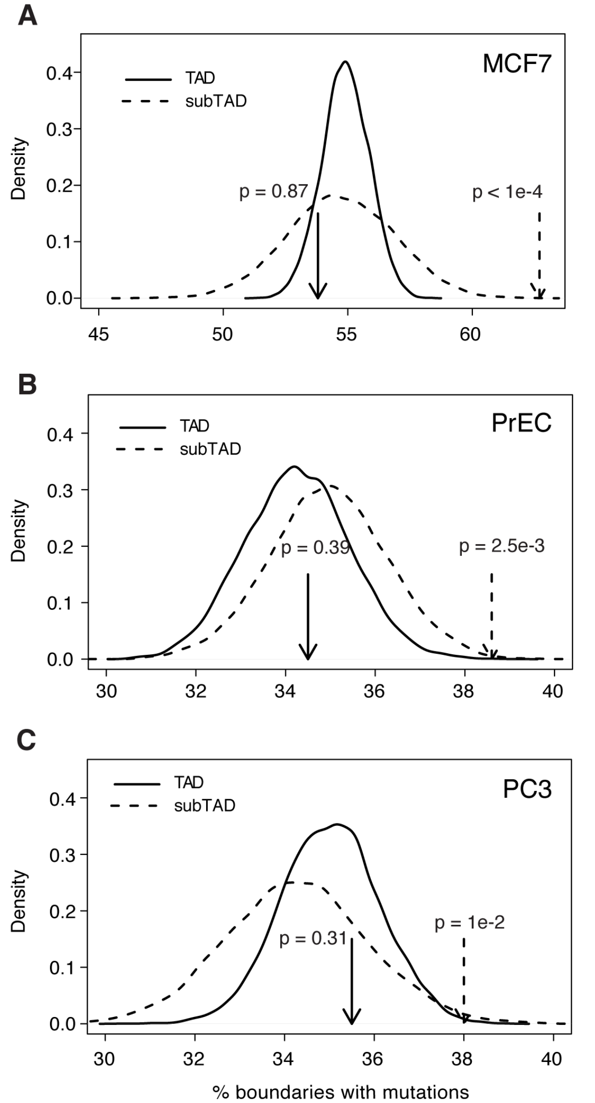

Supplementary References

1. Servant, N., Varoquaux, N., Lajoie, B.R., Viara, E., Chen, C.J., Vert, J.P., Heard, E., Dekker, J. and Barillot, E. (2015) HiC-Pro: an optimized and flexible pipeline for Hi-C data processing. *Genome biology*, **16**, 259.
2. Dixon, J.R., Selvaraj, S., Yue, F., Kim, A., Li, Y., Shen, Y., Hu, M., Liu, J.S. and Ren, B. (2012) Topological domains in mammalian genomes identified by analysis of chromatin interactions. *Nature*, **485**, 376-380.
3. Rao, S.S., Huntley, M.H., Durand, N.C., Stamenova, E.K., Bochkov, I.D., Robinson, J.T., Sanborn, A.L., Machol, I., Omer, A.D. and Lander, E.S. (2014) A 3D map of the human genome at kilobase resolution reveals principles of chromatin looping. *Cell*, **159**, 1665-1680.
4. Imakaev, M., Fudenberg, G., McCord, R.P., Naumova, N., Goloborodko, A., Lajoie, B.R., Dekker, J. and Mirny, L.A. (2012) Iterative correction of Hi-C data reveals hallmarks of chromosome organization. *Nature methods*, **9**, 999-1003.
